# Risk of infection due to airborne virus in classroom environments lacking mechanical ventilation

**DOI:** 10.1371/journal.pone.0314002

**Published:** 2024-11-22

**Authors:** Alexandra Goldblatt, Michael J. Loccisano, Mazharul I. Mahe, John J. Dennehy, Fabrizio Spagnolo

**Affiliations:** 1 Biology Department, Queens College of The City University of New York, Flushing, NY, United States of America; 2 The Graduate Center of The City University of New York, New York, NY, United States of America; 3 Department of Life Sciences, Long Island University Post, Brookville, NY, United States of America; Satyawati College, University of Delhi, INDIA

## Abstract

The COVID-19 pandemic highlighted the role of indoor environments on disease transmission. However, our understanding of how transmission occurred evolved as the pandemic progressed. Enclosed spaces where pathogen-laden aerosols accumulate were strongly linked to increased transmission events. Most classrooms, particulalry in the U.S., do not have any mechanical ventilation systems but do have many people congregating indoors for long periods of time. Here we employ a safe, non-pathogenic surrogate virus, the bacteriophage phi6, to interrogate aerosol transmission in classroom environments that do not have any natural or mechanical ventilation in order to provide baseline understanding of how effectively aerosols facilitate new infections. We measure exposure risk using a modified passive monitoring technique compliant with applicable standards, including ISO 14698–1:2003. We find that virus-laden aerosols establish new infections over all distances tested within minutes and that the time of exposure did not change transmission rate. We further find that relative humidity, but not temperature nor a UV-based disinfection device, significantly lowered transmission rates. Our data suggest that, even without mechanical ventilation, relative humidity remains an inexpensive and highly effective mitigation strategy while UV air treatment may not.

## Introduction

The COVID-19 pandemic refocused attention on the need to understand and mitigate airborne transmission of viruses. The continued fluctuation in COVID-19-associated hospitalizations as well as the added concern over H5N1 avian influenza highlight the ongoing relevance of continuing this work. Originally, transmission of SARS-CoV-2, the virus that causes COVID-19, was thought to occur mainly through coughing, person-to-person contact, and fomites [1]. But, as our knowledge and understanding of both the virus and the disease increased, aerosol transmission emerged as the dominant form of SARS-CoV-2 spread [2–4]. Airborne viruses are believed to predominantly exist inside aerosolized particles of moisture emitted from the upper respiratory tract of infected persons through not only coughing, but speaking [3], singing [5], and even breathing [6]. Coughing is known to produce many larger, heavier droplets (diameter >10 μm) [7], while speaking, singing, and breathing are more likely to produce aerosols (defined here as diameter <10 μm) [3]. Previous studies have shown that the duration of particle suspension in the air is a function of particle size; larger droplets fall out of the air quickly, whereas aerosols can remain suspended for longer periods [8].

Indoor environments, such as gyms, restaurants, schools, or public transportation, have proven to be conducive to viral airborne transmission [5, 9–11]. Data suggests that controlled ventilation (such as HVAC) can substantially lower transmission risk [9]. Likewise, observations support the hypothesis that the risk of airborne virus transmission of pathogens such as SARS-CoV-2 is lower in school environments than in the general community when mitigation procedures, such as mask wearing, are strictly followed [12]. In England, cases were observed to increase with the return of students to in-person instruction [13]. Even under stringent mitigation measures, in-school transmission was still found to be the cause of at least 5% of new cases among those in school buildings [12].

In-school mitigation protocols have become greatly relaxed or even completely eliminated in many areas [14]. However, a lack of mitigation measures may become problematic in the event of either renewed infections or new outbreaks since the majority of classrooms in US schools have insufficient levels of ventilation to provide for clean, safe indoor air [11, 15]. Understanding the risk of exposure and transmission of not just SARS-CoV-2 but other airborne viruses in classrooms with no ventilation, therefore, is likely of high importance, both as a direct application of transmission risk and as a baseline to which we can compare other mitigation tests. Here, we report on an investigation into the potential for exposure in unventilated classrooms using a live, aerosolized, airborne virus incapable of infecting human or animal cells. This bacteriophage (or simply “phage”) is a virus that only infects a bacterial host and is therefore safe for use around humans. We expose susceptible host cells to this aerosolized virus and quantify the number of transmission events [16] over time using an exceedingly simple and reliable modified passive monitoring technique. We then introduce some mitigation strategies that may improve the health of the classroom’s occupants and quantify their effects.

## Results

Results were found to be consistent with a previous study employing the same basic experimental design [17], even without the mechanical ventilation used in that study. The relative exposure rate reported here is normalized to 4 × 10^7^ total phage released per trial so as to standardize results and to account for any variation in the amount of phage aerosolized across the numerous trials. This normalized viral load is consistent with the range of reported observations for SARS-CoV-2 [18]. We define relative exposure rate as the rate of transmission per unit time (in 15-minute blocks) normalized to 4 x 10^7^ total phage aerosolized at any given distance. This relative exposure rate is in line with the recommended practices for passive air sampling found in EN ISO 14698–1:2003 [19], since active air sampling would be inappropriate in this situation [20] and fail to provide quantifiable data. Exposure is measured by counting the number of blue viral plaques on an exposed pertri plate, each of which represent successful aerosolization, airborne travel, transmission, and subsequent infection by our genetically marked, enveloped phi6 into a complementary, genetically-modified bacterial host cell.

**Fig 1:**
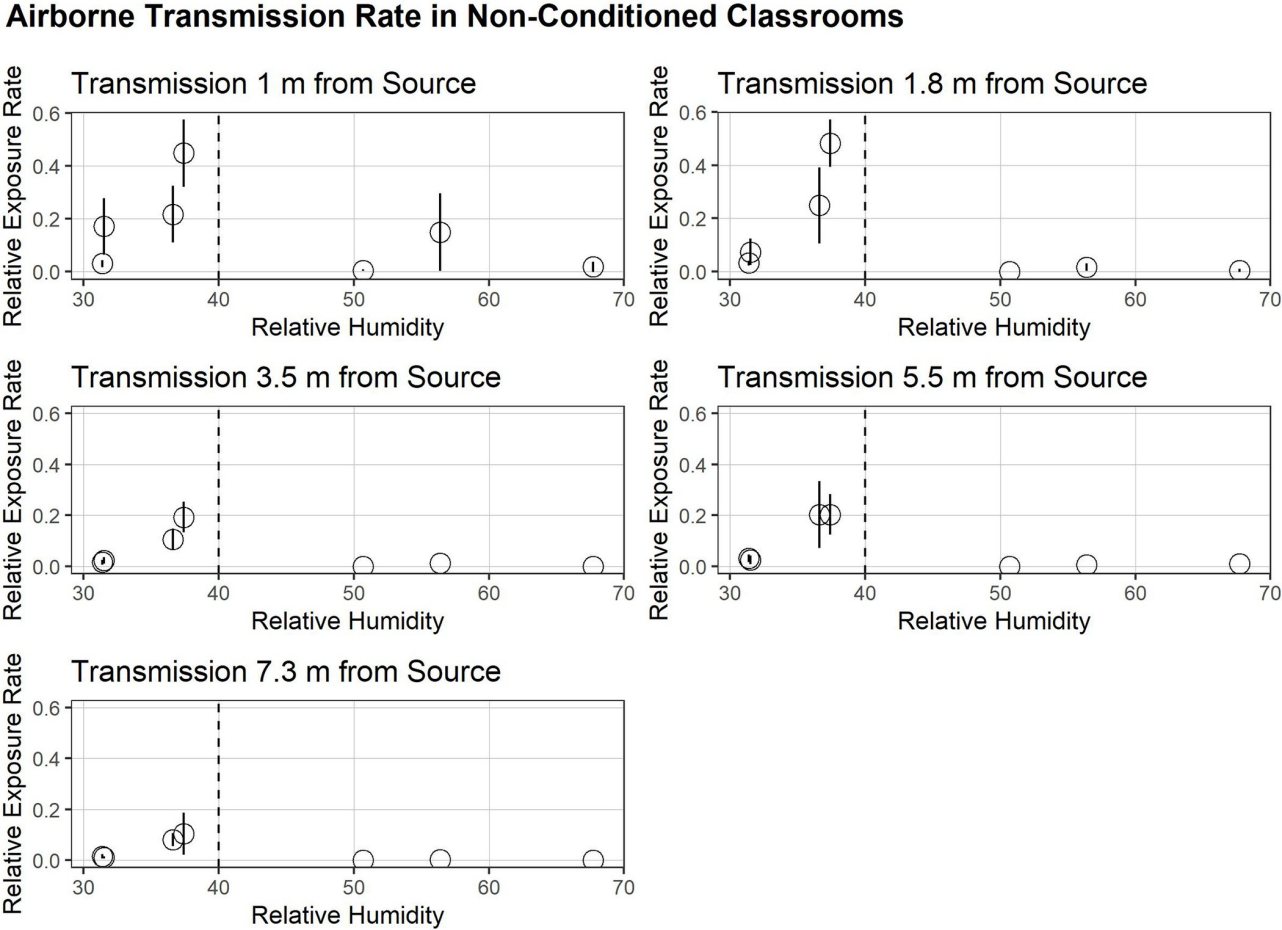
Airborne transmission rate in non-conditioned classrooms. Relative exposure rates represent the rate of exposure per unit time (in 15-minute blocks) normalized to 4x 10^7^ total phage aerosolized in order to allow for comparison of multiple trials with variability in total number of phages aerosolized. At all distances we observe a drop off in the relative exposure rate when RH > 40%. Relative exposure rates are empirically higher within 1.8 m (6 ft) of viral source. Error bars represent 95% confidence intervals.

Our results indicate that there was a statistically significant increase in the exposure rates when relative humidity (RH) was below 40% when compared to RH higher than 40% (p value = 5.45 x 10^−7^, two-tailed T-test). Although plates closer to the sources at 1 and 1.8 m (3 and 6 ft, respectively) had higher overall exposure rates compared to the plates further away from the virus source, no additional effect by distance was observed. Instead, a strong and consistent trend of exposure rates dropping at RH greater than 40% was observed, at all distances measured, up to 7.3 m (24 ft) away from the source (Fig 1).

**Fig 2:**
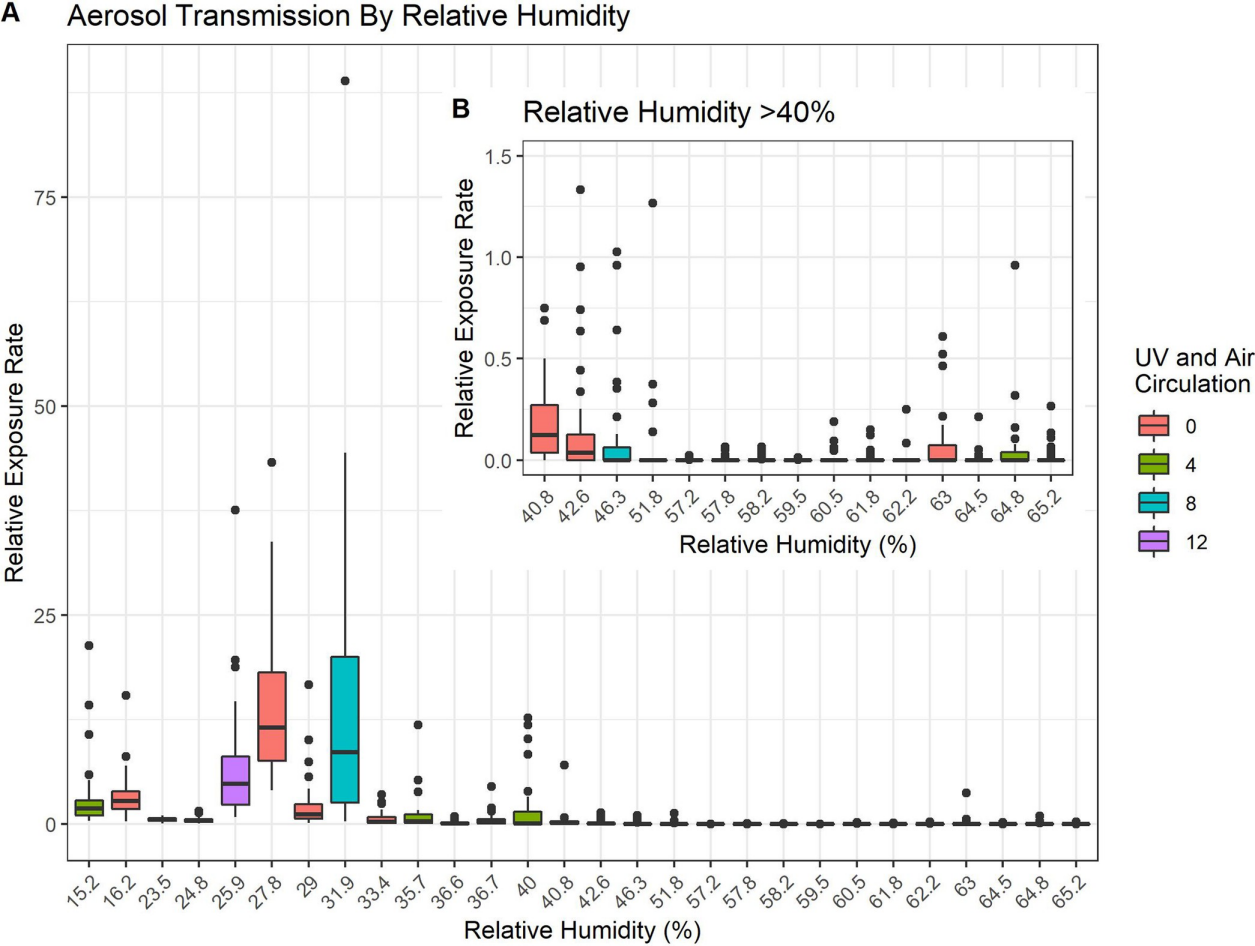
Aerosol transmission by relative humidity. Relative Exposure Rates for all Trials. A. The relative exposure rates for all trials are arranged by RH. A drop in transmission is evident from RH ≥ 33.4%, however, there is a high degree of variability until RH ≥ 40.8%. B. The inset panel represents all trials with RH > 40% with the y-scale changed for easier viewing. Color indicates any UV treatment/fan speed. Red bars are no UV control conditions, green bars are UV treatment with “low” fan settings, blue bars represent UV treatment with “medium” fan setting, and purple bars show UV trials with fans set to “high” speed.

**Fig 3:**
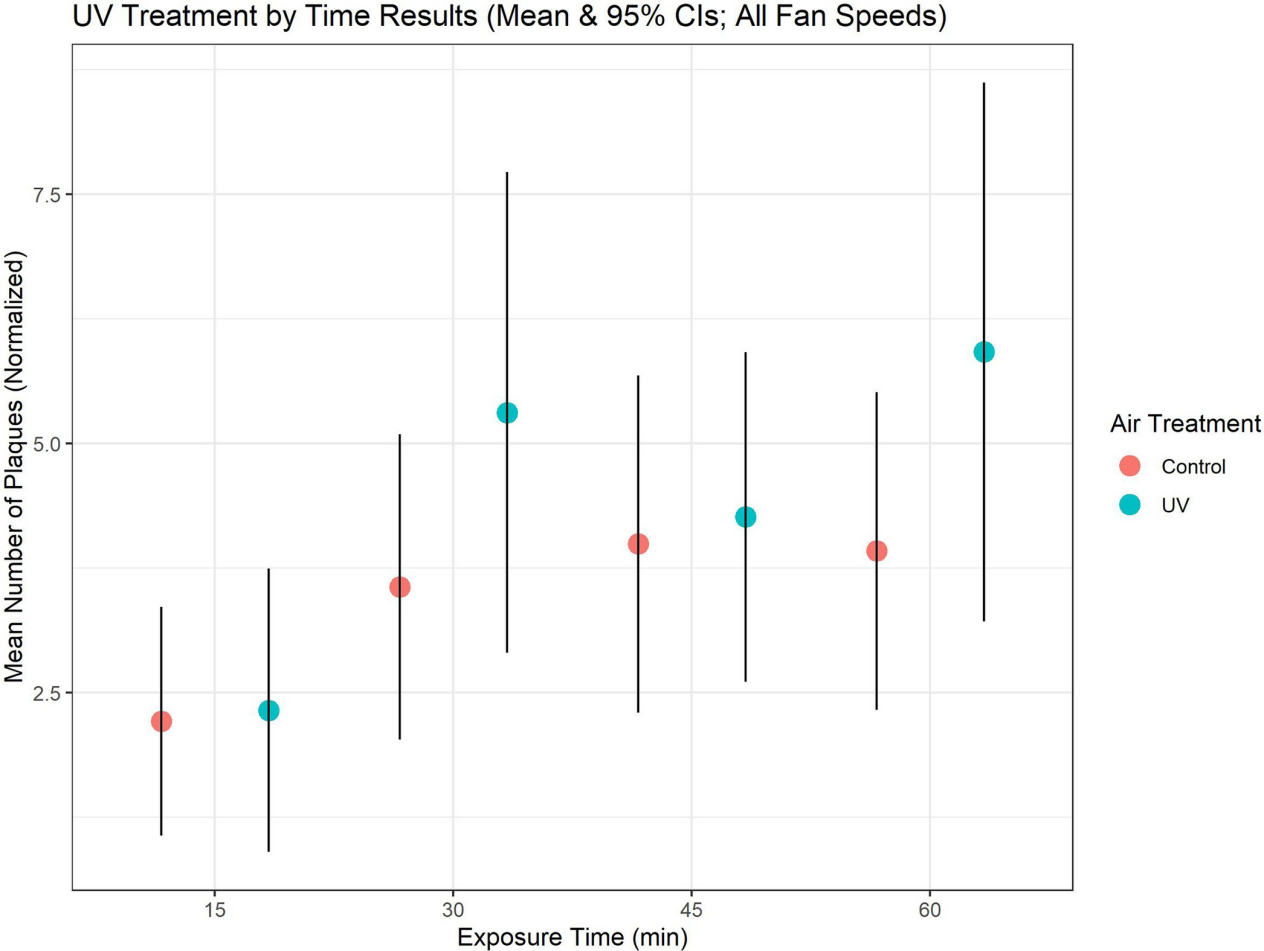
UV treatment by time. Mean number of phage plaques (normalized) by time of exposure and UV treatment. Attempts to disinfect the classroom air through use of portable UV-C generating devices had no effect upon the number of transmission events observed, regardless of the UV device fan speed or the amount of time of UV treatment. Some evidence suggests that the specific type of airborne virus as well as the wavelength of UV light along with various environmental conditions could affect disinfection results [21], although no impact was observed with the units used in this study.

The same two laboratory-style classrooms were used for investigating the effect of commercially available portable UV air disinfection units, with one room acting as a control and one containing the UV fan unit(s) (Arc Air, R-Zero). In rooms with the UV treatment, we did not see a statistically significant impact of UV disinfection on the mean number of plaques observed during matched periods of exposure, regardless of the speed at which fans were operated (Fig 2). In fact, there was a slightly higher mean number of plaques in the UV treated rooms compared to the control (Fig 3), although this effect was not statistically significant.

Across all fan speeds, relative humidity remained the most significant factor observed. RH over 40% within UV trials have relative exposure rates below 1.5 on average. The maximum exposure rate within this group is 4, which is still extremely low compared to matched exposure rates when RH was below 40%.

## Discussion

Our results support the hypothesis that aerosolized viruses can and do transmit in indoor environments over long distances (up to 7.5 m or 24 ft) *within minutes* of their release, although the overall number of transmission events is reduced by half over such distances. This result is important as a baseline understanding of aerosolized pathogen spread in planning for any mitigation of airborne infectious diseases in the built environment, especially considering that these are the places where humans now spend a majority of our time [22]. In particular, locations such as classrooms pose a potential risk as the numerous people present spend long periods of time together in these rooms, often without mechanical ventilation systems or sources of fresh air [23]. Mitigation strategies to limit indoor transmission, then, become important, particularly since many such rooms have low rates of air exchange.

We find that the most consistent environmental mitigation is also one of the simplest and involves maintaining relative humidity above 40%. This RH level is not extreme or even uncomfortable for human or animal inhabitants. In addition, equipment capable of achieving and maintaining a RH of 40% is neither particularly expensive nor difficult to install or run and can easily be added to a room environment without greatly impacting the designated activity in the space. Relative humidity could be effectively maintained in a classroom using one or more portable humidifiers as needed and to suit the particular room. These are unlikely to drastically add to noise levels or provide any unwanted level of distraction. In seasonal climates, warmer months are typically associated with higher RHs naturally and may not even need humidity added (as was the case in our experimental classrooms). In cooler seasons, artificial (and modular) humidifiers can be put in place for a fraction of the investment cost required to upgrade mechanical ventilation.

Additional potential *ad hoc* mitigation measures include using UV-C light as a disinfectant. In our testing *in situ*, a portable commercially available UV disinfection unit did not impact exposure risk to the same extent as RH, even when multiple units were simultaneously used in the same room. This result remained consistent regardless of the fan speeds tested; no fan speed proved to be more effective than the control room tested contemporaneously without the UV units. Importantly, results observed for UV mitigation here may not be representative of UV-based disinfection approaches or their efficacy, as device design, location, performance, and application vary widely [21].

In the U.S., mechanical ventilation standards for schools require minimum rates of fresh air per occupant per second [24]. However, most U.S. schools fall far short of the minimum standards for mechanical ventilation, with associated losses in student performance [23]. The most direct solution to this issue, and the one likely to have the most imapct, would be to upgrade school ventilation systems to meet the standards. This would, we acknowledge, be a costly and time consuming undertaking. As such, low-cost mitigation techniques are needed. Baseline understanding of transmission rates in school rooms without mechanical ventilation is necessary in designing effective mitigation strategies.

Here, we investigated airborne viral transmission in non-ventilated classrooms and found that successful infection events were not dependent upon distance or total time of exposure, although longer exposure time did lead to higher variability in transmission. Instead, the strongest and most consistent factor we observed in the rate of transmission was relative humidity. Our data indicate that controlling RH in classroom environments could potentially be an effective and achievable mechanism to mitigating airborne viral transmission. In addition, humidity management and monitoring are low cost and can be done on an *ad hoc* basis in classroom environments, with little upfront costs or loss of space utilization. The threshold indicated for effective airborne pathogen mitigation is 40%, which is within the range of comfortable indoor environments for people, plants, animals, and equipment. Portable humidifiers can be used to help achieve the 40% RH target, with the added benefit that they can be shut down or moved when not in use. As a mitigation strategy, RH looks to be broadly applicable, low-cost, easily applied, and highly effective for classroom environments.

### Study limitations

While the results presented here support the conclusion that transmisison risk for our airborne phi6 phage is reduced when RH climbs above 40%, we note that our experimental virus is a proxy and not an actual human pathogen. Phi6 has been used as an experimentally tenable proxy for SARS-CoV-2 in previous reports [17, 25]. However, modeling studies [26, 27] suggest that airborne pathogens, such as SARS-CoV-2, influenza, and adenovirus, are sensitive to specific biological and environmental parameters capable of impacting the rate of decay in airborne droplets and aerosols.

Likewise, the results presented here concerning UV treatment may not be broadly applicable for human pathogens. UV systems, in general, are not optimized for non-human pathogens, such as phi6 and our surrogate virus may be particularly resilient to the potential impacts of UV. In addition, the range of UV-treatment approaches available made testing a representative sample impracticable.

## Materials & methods

### Experimental design

In a manner similar to Skanata and colleagues [17], we used a simple experimental setup to investigate potential asymptomatic aerosol transmission in built environments using a genetically modified *LacZ-****β***-marked phi6 bacteriophage as a proxy for viruses such as SARS-CoV-2 [17, 25, 28]. Phi6 is a lipid-membraned enveloped phage that has similar size, shape, and physiological characteristics to SARS-CoV-2, but is not pathogenic and cannot infect humans, allowing for ease of testing in built environments. Phi6 does, however, reproduce within and ultimately kill the susceptible bacterial host, *Pseudomonas phaseolicola*, in much the same way that viruses such as SARS-CoV-2 [29] or influenza [30] kills human cells. We used a measured amount of live virus (set to match relevant levels of airborne SARS-CoV-2 [18]) and mechanically aerosolized this pathogen. We then quantified the number of successful infections of host cells growing on petri plates set distances away (easily identified by their blue plaques). Here, we defined transmission as the combination of live virus becoming aerosolized, moving through the room air, finding a host cell, successfully infecting that host cell, and killing it.

### Host preparation

A single colony of LacZ-**α** producing *Pseudomonas phaseolicola* [28] was added to 30mL of lysogeny broth (LB) containing 30 mg of ampicillin and incubated for 18 h with rotary shaking (220 rpm) at 25°C to produce an overnight culture for soft agar overlay plates. A susceptible bacterial lawn was created using 200 μL of the overnight culture in 3 mL of soft agar to serve as “detectors” of viral infection for the experiment.

When *LacZ-****β***-marked phi6 infects and kills susceptible LacZ-**α** producing *P*. *phaseolicola* host cells, viral plaques are produced. When this infection, killing, and plaque production happens using the genetically engineered LacZ-**α*β*** system and the host is grown on LB agar supplemented with X-Gal, the resulting plaques appear blue [28]. This use of *LacZ* complementation between phage and host ensured that any blue plaques observed on test plates resulted from aerosolized experimental phage and not from any possible phage that happened to be naturally occurring in the room.

### Phage preparation

To prepare our phi6 experimental lysate (experimental viral pathogen), 5 mL of stationary-phase *P*. *phaseolicola* (host) overnight culture was added to 200 mL fresh LB. When the culture reached exponential phase,10 μL of frozen phage stock was added. Following 18 h incubation with shaking, phages were isolated by filtration through 0.22 μm syringe filters (Durapore; Millipore, Bedford, MA). Phage particles per mL (titer) in the phage lysate were quantified via serial dilution and plating according to standard methods [31].

### Generation of aerosols

The experiments used medical delivery nebulizers (UniHEART nebulizer, Westmed, Inc, Tucson, AZ) connected to an air compressor (Model 0399, Westmed, Inc, Tucson, AZ) to generate aerosolized viral droplets (MMAD 2–3 μm) at a rate of 6 L/min, similar to human respired volumes (for more detailed explanation see SI) [32]. To introduce phi6 into a classroom environment, the viral lysate was diluted in LB to 10 mL and inserted into the nebulizer. The diluted phage lysates had a concentration of approximately 10^8^ phage particles per mL (maximum viral load of experimental room per trial = 10 mL x 10^8^ phage/mL = 10^9^ phage per trial).

### Detection methodology

While there is no universally acknowledged way to detect airborne biocontaminants, most procedures fall under the headings of either active or passive sampling [19, 20]. Active sampling involves an impeller of some sort channeling air into or onto a test device, usually an agar plate [19]. In a preliminary test of a direct impaction air sampler that collected a large volume of virus-laden room air and impacted those virons onto an agar plate, the number of individual viral infections was not quantifiable because too many plaques were occurring in too small an area on the agar plate. This caused larger blue conglomerated plaques instead of small, countable plaques resulting from individual infections.

Likewise, a genomic approach would not be applicable to this investigation. While viral genomes can be easily detected from a sample, even one taken from the air, a quantitative measure of the number of genomes would not accurately represent the number of viable virus in the sample, as genomic material quantified using molecular techniques, such at RT-qPCR [33], can long outlast the ability of a viron to infect a host. Such misapplied techniques resulted in a delayed understanding of SARS-CoV-2 transmission dynamics early on in the pandemic as viral RNA was found on everything from the mail to groceries.

For these reasons, as well as a desire to have the methodology as broadly applicable as possible, a passive approach was used. We modified the classic settling plate approach such that agar plates with living host cells were arranged vertically (i.e., perpendicular to the ground) at heights from the floor comparable to where the noses and mouths of potential students would be (See SI, S3 & S4 Figs). This allowed us to: 1. quantify the number of infection events per unit time at various distances from a source, 2. Measure only those viruses suspended in the air at specific distances (as opposed to those falling out of the air due to gravity), and 3. Test the effects of environmental factors, such as relative humidity (RH) or UV treatment.

### Detection of aersolized virus in classrooms

Standard petri plates containing LB agar supplemented with X-Gal and ampicillin were inoculated with the genetically modified strain of *P*. *phaseolicola* producing LacZ-**α**. These plates were placed vertically at varying distances from the nebulizer for up to 60 minutes of exposure. Both the nebulizer’s aerosol port and the detector plates were placed at an approximate height of the nose and mouth of students that might have been seated in the classroom (S4 Fig). This design simulates a situation where an unmasked individual is spreading airborne viruses in a classroom full of students. The plates were located 1, 1.8, 3.5, 5.5, and 7.3 meters (3, 6, 12, 18, and 24 feet respectively) away from the nebulizer to detect airborne transmission of phi6 (S3A & S3B Fig). Two sets of four plates were placed on the left and right sides with respect to the nebulizer at each of the different distances. Each plate within a set was exposed to the aerosolized phage for progressively longer durations of time for up to 60 minutes (i.e., 15, 30, 45, and 60 min). Traffic within the rooms was limited to the experimenter and even then only at the end of each 15 minute interval in order to cover petri plates as needed. All rooms had covered control plates to verify that arsolized virus could not infect covered plates.

Experimental trials were repeated over multiple days in two different non-ventilated rooms with the same dimensions and configurations at CUNY Queens College in New York City. These test rooms were located on different floors of the same building and are typically in use during semesters for instruction. There is no mechanical ventilation in these rooms and the only passive ventilation is windows, which were closed during all trials. This provides a real-world example of non-ventilated classrooms. Experiments were conducted on days with low humidity (<40% relative humidity) and high humidity (>40% relative humidity) under naturally occurring temperature conditions. Room temperature and relative humidity were continuously monitored using wireless sensors (Model H5074, Govee, Shenzhen, China).

### Classroom experiments with UV

An identical experimental setup was conducted in the same non-ventilated rooms with the presence of portable ultraviolet (UV) fan units (Arc Air, R-Zero, Model: RXAIR) supplied by CUNY Queens College for all UV trials. These UV units were designed to take in and disinfect air in occupied rooms, as opposed to units that use more powerful UV light sources to disinfect air and surfaces in unoccupied rooms. Air is brought into the unit by fans, in a manner similar to HEPA or Corsi-Rosenthal Boxes [34]. For UV trials, one of the two nearly identical test rooms would have two UV units, placed diagonally across from each other in the two corners of the room. The other room served as the control condition. In subsequent trials, the experimental room was used as a control room and *vice versa*. Data was collected under both low humidity (<40% relative humidity) and high humidity (>40% relative humidity) conditions. The UV units have 16 fan speeds. We tested at least three replicates each in the low (fan speed = 4), medium (fan speed = 8), and high (fan speed = 12) fan speeds.

### Experimental titer

Plates were collected at the end of the experiment and placed in an incubator at 25°C. The volume of lysate remaining in the nebulizer at the end of the experimental trial was collected to determine the total volume of phage aerosolized. Titer of the experimental lysate was reconfirmed via plating after all trials. In 24 to 48 hours, infection of the *Pseudomonas* bacteria by marked phi6 in aerosolized particles resulted in blue plaques on the X-Gal containing medium [35]. The number of successful transmission and infections was then counted and the data recorded.

## Supporting information

S1 Appendix (PDF)

S1 Fig Correlation between room temperature and relative humidity. (TIF)

S2 Fig Transmission by temperature at each distance tested. (TIF)

S3 Fig Experimental setup in classrooms without mechanical ventilation. (TIF)

S4 Fig Diagram of basic experimental setup. (TIF)
