# Supplementary material for: Risk of infection due to airborne virus in classroom environments lacking mechanical ventilation
Source: PLoS One. 2024 Nov 22;19(11):e0314002. doi: 10.1371/journal.pone.0314002 (PMC11584072; doi:10.1371/journal.pone.0314002)
Supplement: S1 Appendix — (PDF) [file pone.0314002.s001.pdf]

SUPPLEMENTAL INFORMATION FOR

# Risk of Infection Due to Airborne Virus in Classroom Environments Lacking Mechanical Ventilation

Alexandra Goldblatt, Michael J. Loccisano, Mazharul I. Mahe, John J. Dennehy, & Fabrizio Spagnolo

## Transmission:

Throughout this study, we define transmission as a biologically relevant result or illness following exposure to a pathogen originating from an infected person, an infected animal, or a contaminated object. This definition is in accordance with the standard understanding of transmission in an epidemiological context (1).

## Rate of Aerosolization:

The average volume of a human breath is 408 ml (2) and typical respiration rate is 18 breaths/minute. At these values, a human exhales 440 liters per hour on average, or 7.3 liters per minute. This rate is similar to the 6 liters of air per minute passing through the nebulizer used in the experiments described here.

Supplemental Figures:

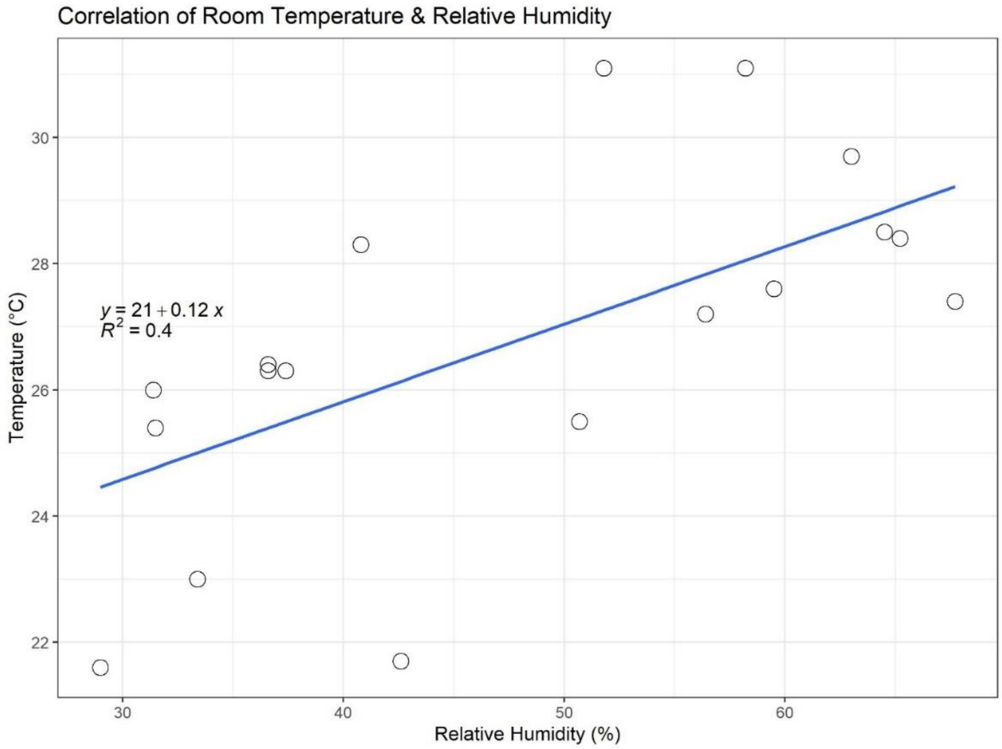


Figure S1: Correlation between Room Temperature and Relative Humidity: The average room temperature and RH are reported for all experimental and control trials. While RH generally correlated with temperature, the correlation was not strong ($R^2$= 0.4). In general, RH increased as temperature increased. This pattern is typical in most climates. The effect on transmission was found to be a result of RH and not temperature (see Fig. S2).

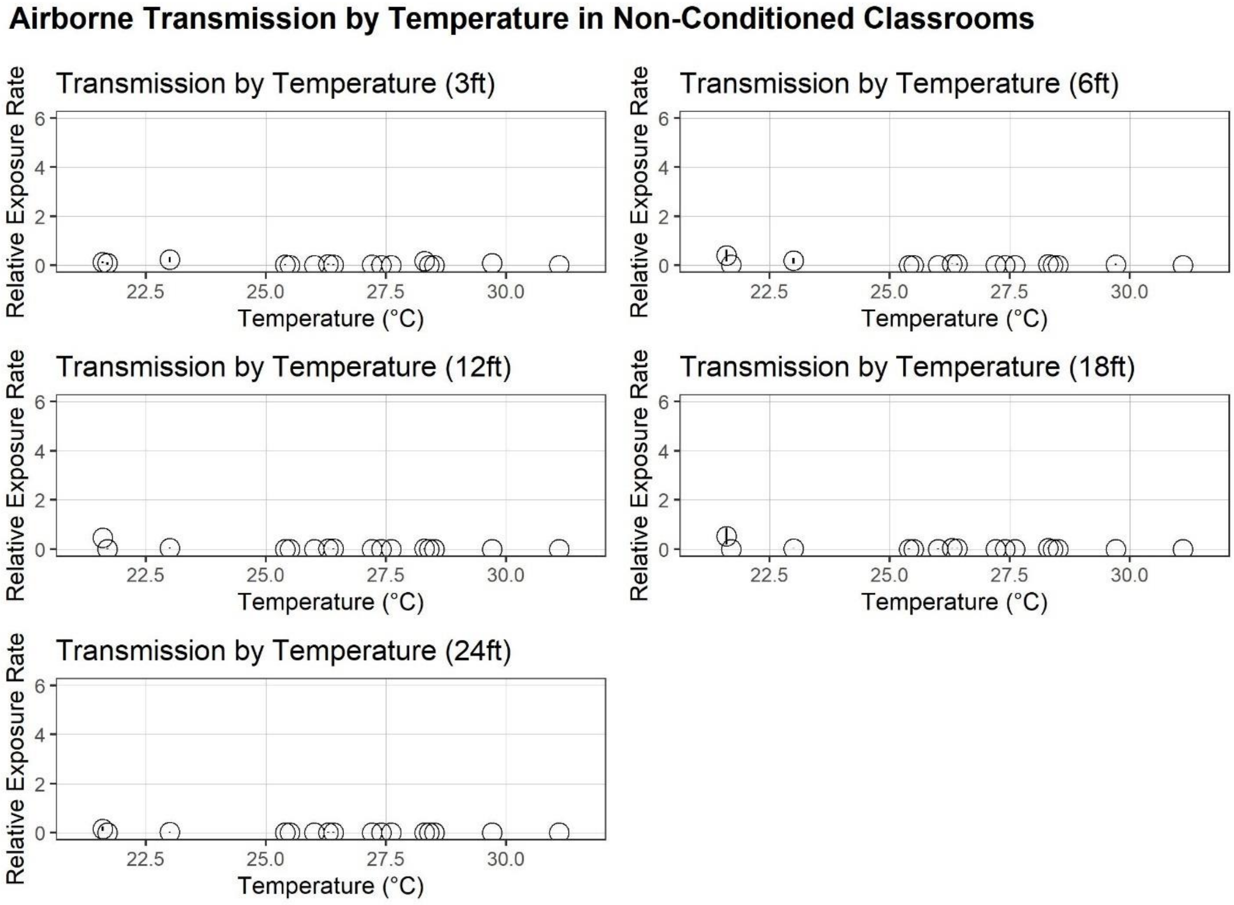


Figure S2: Transmission by Temperature at Each Distance Tested: Average room temperature did not show a correlation with relative exposure rate. Note that scale of Y-axis is set to match that in Figure 1.

A B

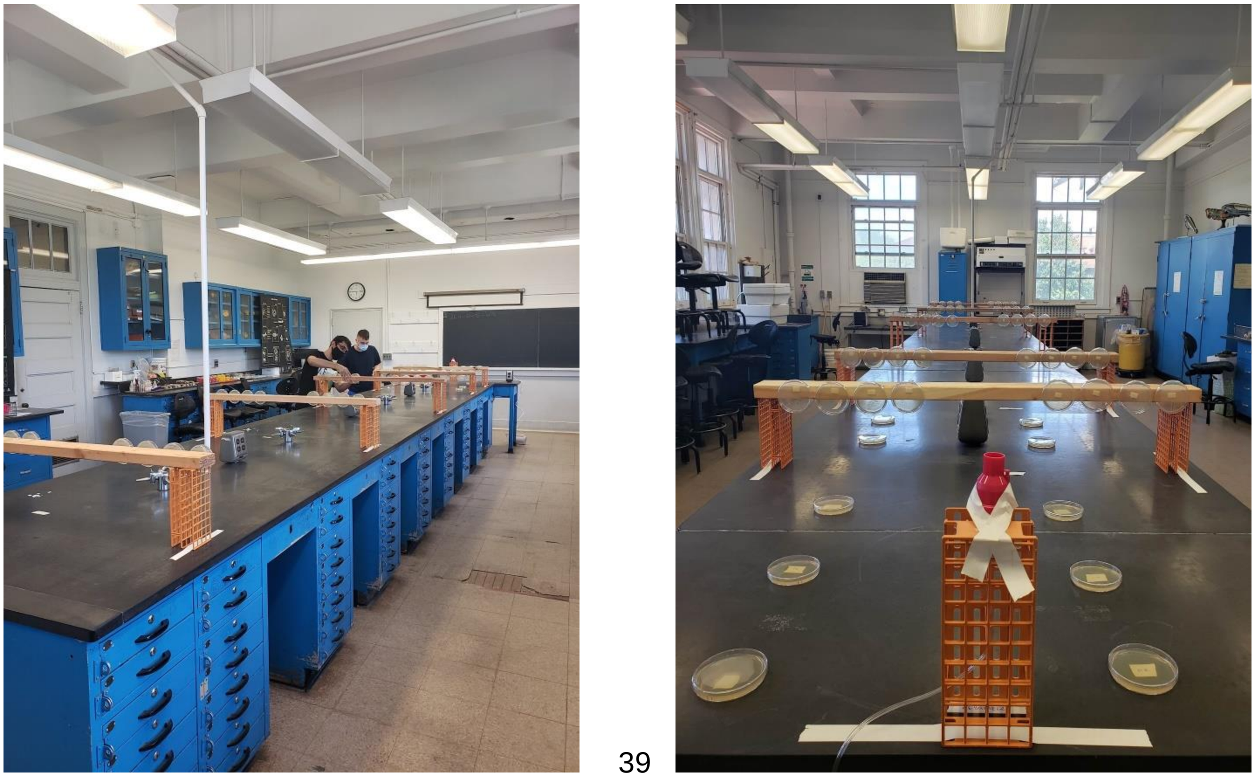

Figure S3: Experimental Setup in Classrooms Without Mechanical Ventilation. A. Experimenters set up petri plates seeded with lawns of LacZ-**α** producing *P. phaseolicola* on LB agar. Plates were installed vertically on both the left and right sides of the room in rows of 4 on each side, one plate for each block of timed exposure (15, 30, 45, and 60 minutes). B. A medical-grade nebulizer was the source of generated aerosols laden with *LacZ-**β***-marked phi6 bacteriophage (pink device, center foreground). Vertical detector plates were arranged on left and right sides of the nebulizer at 1, 1.8, 3.5, 5.5, and 7.3 meters (3, 6, 12, 18, and 24 feet respectively). Horizontal plates were used near the nebulizer to verify large droplets falling out of the air.

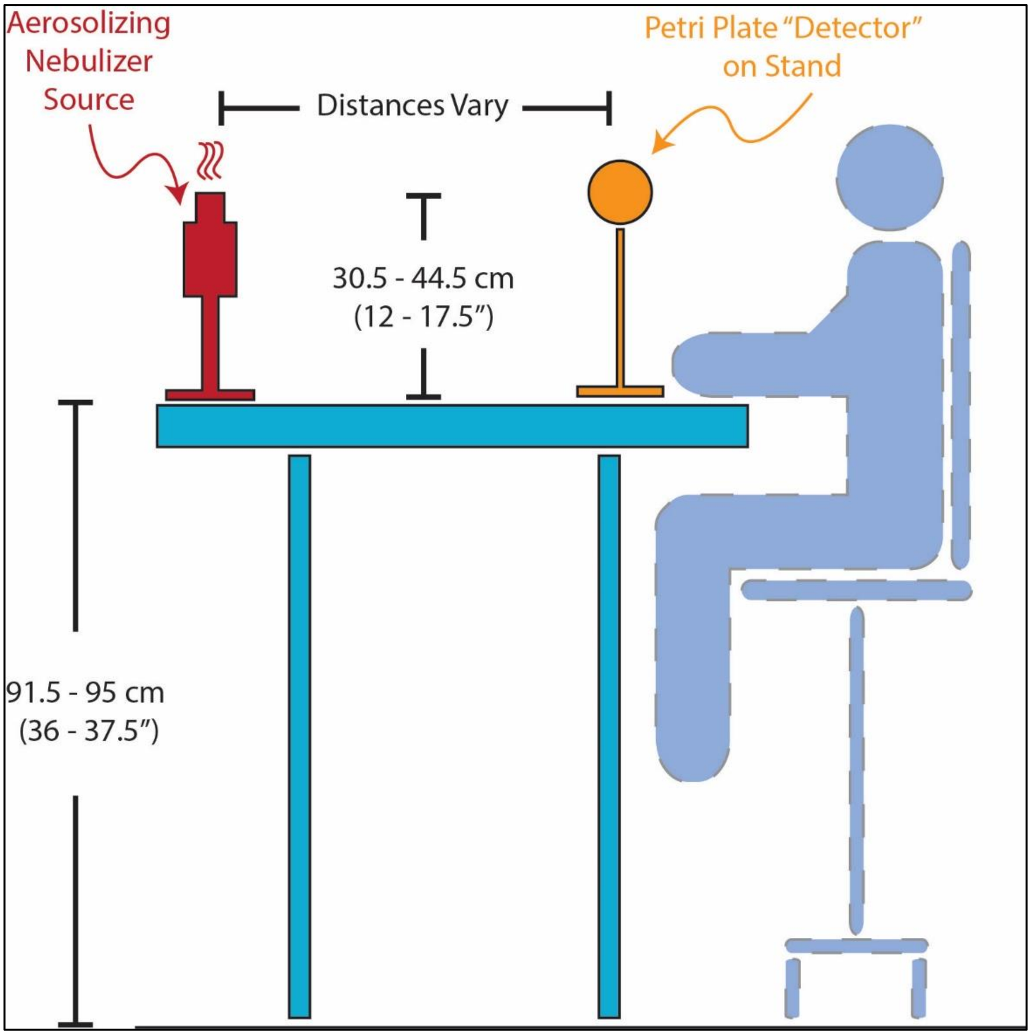


Figure S4: Diagram of Basic Experimental Setup. Petri plate detectors and the nebulizer exit port were set to match the approximate height above the writing/working surface of a student's mouth and nose. In this way, the experimental design mimics the most likely point of contact between aerosolized virus particles and a potential host. Horizontal distances between the nebulizer and the petri plates are as described in the Methods.

References

1. Van Seventer JM, Hochberg NS. 2017. Principles of Infectious Diseases: Transmission, Diagnosis, Prevention, and Control, p. 22–39. *In* Quah, SR (ed.), International Encyclopedia of Public Health, 2nd ed. Elsevier.

2. Gilbert R, Auchincloss JH, Brodsky J, Boden W. 1972. Changes in tidal volume, frequency, and ventilation induced by their measurement. J Appl Physiol 33:252–254.
