# Supplementary figures and images for: Risk of infection due to airborne virus in classroom environments lacking mechanical ventilation
Source: PLoS One. 2024 Nov 22;19(11):e0314002. doi: 10.1371/journal.pone.0314002 (PMC11584072; doi:10.1371/journal.pone.0314002)

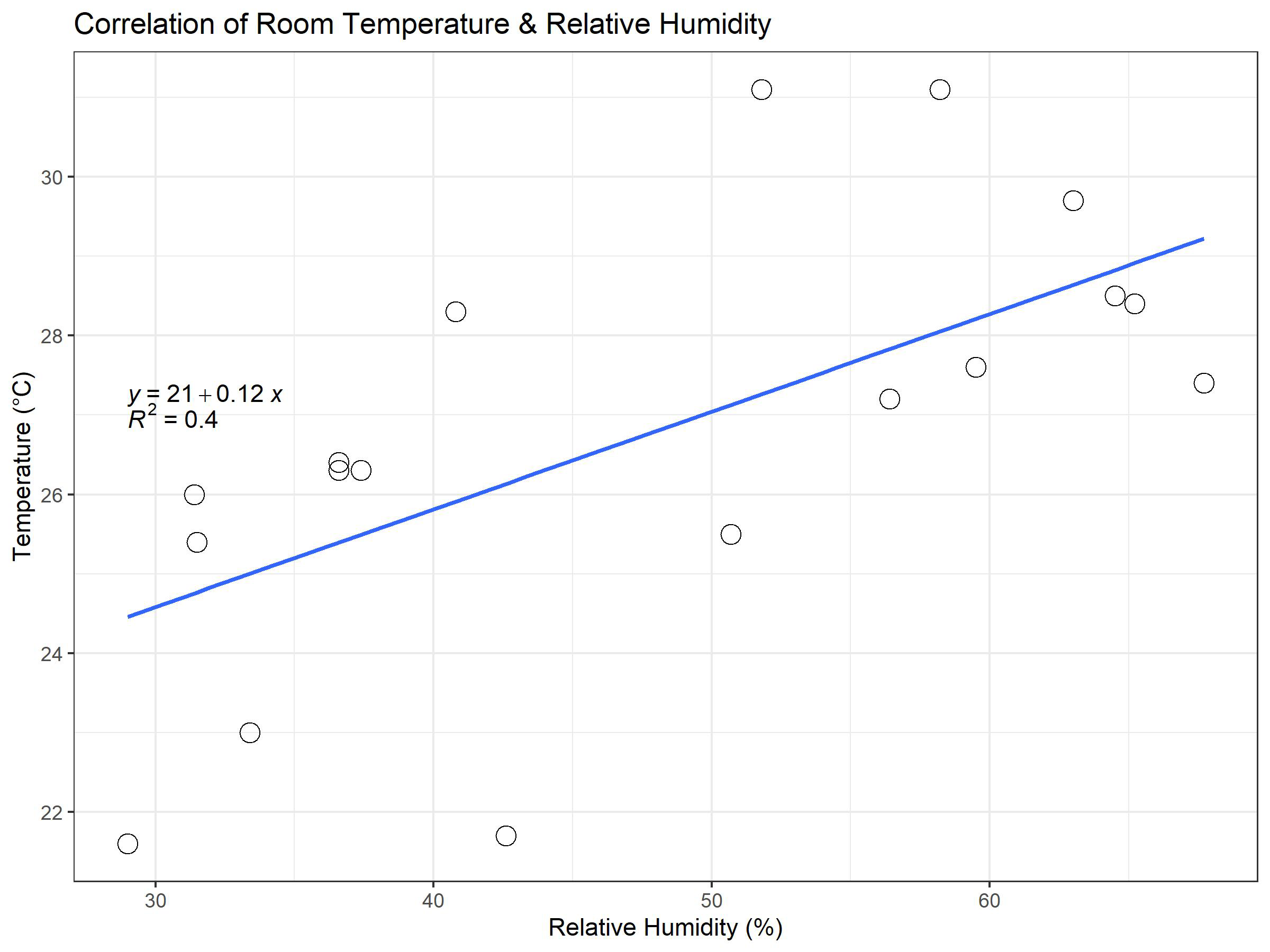

Supplement: S1 Fig — (TIF) [file pone.0314002.s002.tif]

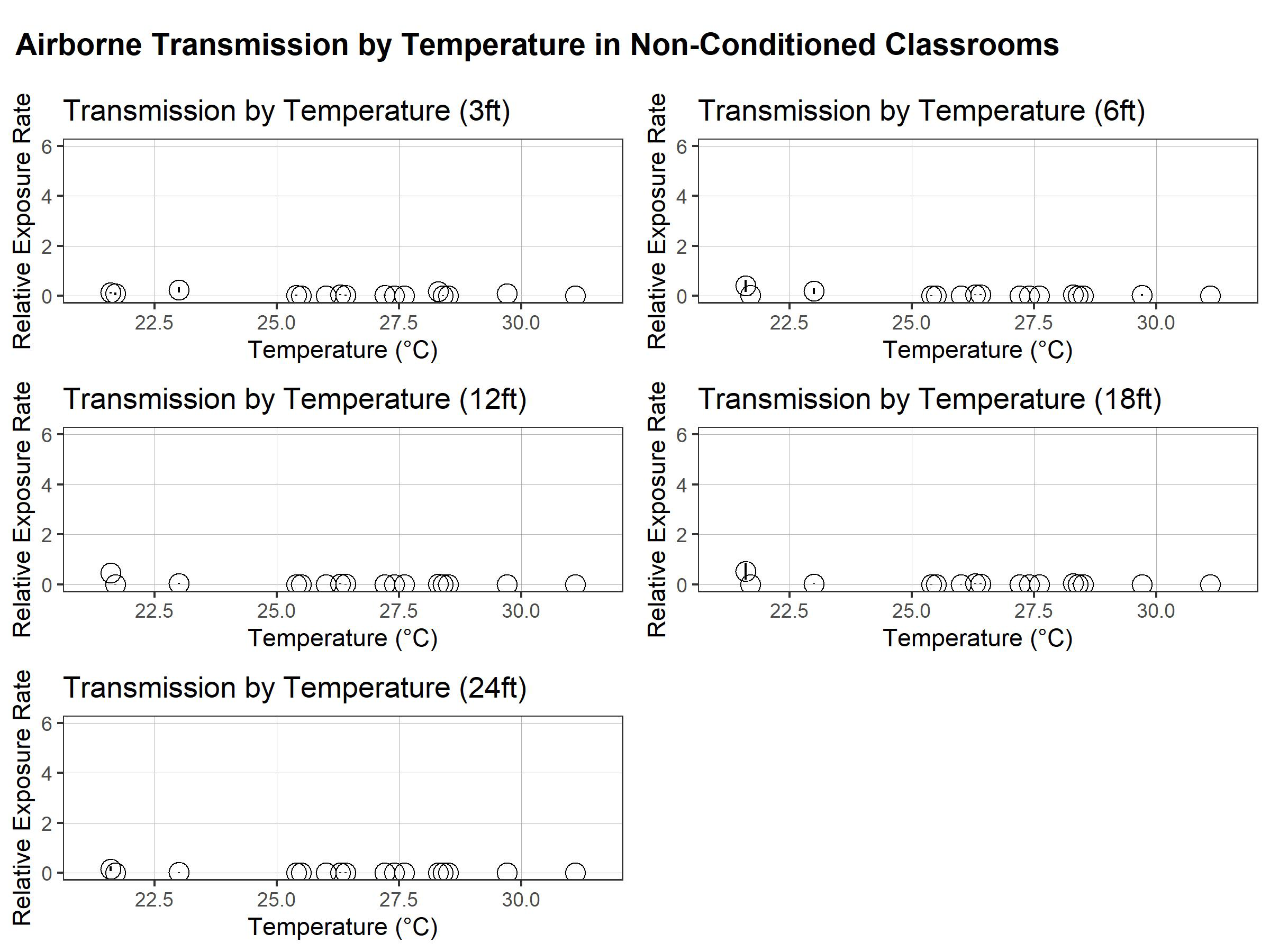

Supplement: S2 Fig — (TIF) [file pone.0314002.s003.tif]

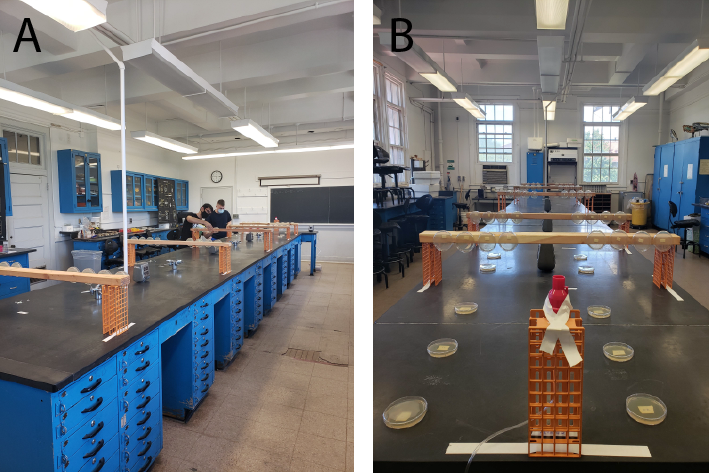

Supplement: S3 Fig — (TIF) [file pone.0314002.s004.tif]

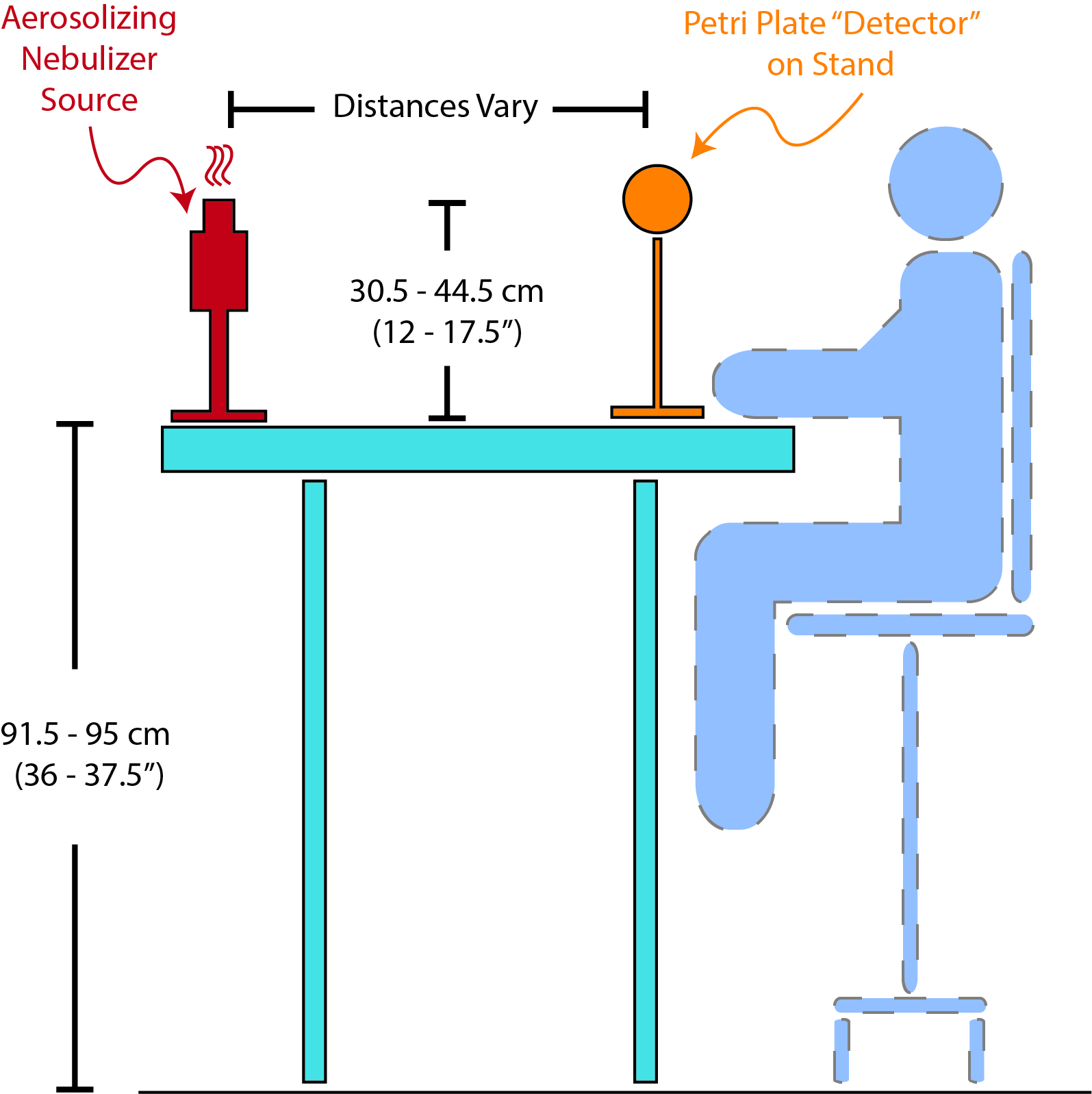

Supplement: S4 Fig — (TIF) [file pone.0314002.s005.tif]
